# The Cattle-tick Plague; Preventive Treatment

**Published:** 1897-02

**Authors:** Cooper Curtice

**Affiliations:** Veterinarian, Moravia, N. Y.


THE JOURNAL

OF

COMPARATIVE MEDICINE AND VETERINARY ARCHIVES.

VOL. XVIII. *FEBRUARY*, 1897. No. 2.

### THE CATTLE-TICK PLAGUE: PREVENTIVE TREATMENT.

By COOPER CURTICE, VETERINARIAN,
MORAVIA, N. Y.

WHILE endeavoring to prevent animals from contracting communicable disease, two plans have been conceived and acted upon. Each of these has been to a degree successful. The more important of these consists in keeping the disease from spreading beyond the animals or place in which it is discovered. The other prepares the systems of individual animals to resist disease by either producing a modified form of the disease in them, or by the injection of material into their veins which is designed to entirely prevent or at least to ameliorate attacks otherwise fatal.

When Jenner taught the medical profession vaccination against smallpox he conferred upon mankind the greatest medical benefit of his time, and rightly became immortalized. Yet, however much credit be due to vaccination in restricting the spread of smallpox, far more is undoubtedly due to the strict quarantine measures which have since been taken in the preventive treatment of the cattle-tick plague officially termed "splenic fever," Southern fever of cattle, or Texas fever by the United States Bureau of Animal Industry. But one of these plans has to any extent been tried by man, viz., the quarantine of the region whence it spreads during the time of year it prevails. The method by vaccinal treatment prevails largely in nature, but to my knowledge has not been intentionally put into practical use outside of a few laboratory experiments, and the custom of one Southern proprietor. The method was first discussed by Dr. Theobald Smith, in his admirable report on Texas fever in 1893.

To which of these methods must cattlemen and veterinarians of the infected districts look for deliverance from the plague?

Immunity of Southern cattle is conferred upon them either when they are calves by a single attack of the disease or more rarely by one or more mild attacks when older.

That not all in the infected area are immunized is evidenced by the large death-rate from this source. So far as is now known, this immunity is conferred by the cattle being naturally inoculated by ticks. The disease inoculated into calves is the same as that which proves fatal to older cattle, the different results depending on some inherent difference between the young and old. The exposure is unintentional and accidental for the most part. It is more certain to occur when ticks are uniformly on all farms, and less where many are entirely free. In the latter places inoculation and death occur most frequently.

Mr. Benjamin W. Hunt, a wealthy planter of Eatonton, Georgia, says in his catalogue of the Panola farm for 1894: "There is a debt owed to the public by persons who have studied Southern cattle-fever. My experience has been so different from all published information on this subject that I ask its careful consideration. In the first place, a calf raised South is no more exempt from its deadly effect than one raised in Canada. Only after the Southern-raised animal has long grazed upon thoroughly infected pasture-land should it be sold South. Further, there are many places in Georgia where whole farms and parts of farms are not infected at all. I can raise a calf at my race-track or about my barns, and when it is sent to my regular creek-bottom pasture it will be attacked as violently with cattle-fever as a Northern-bred animal. I have long practised giving the fever to every milk-drinking calf, and as the disease is non-recurrent and not dangerous to any young bovine animals, my herd is, I believe, absolutely immune. No Southern buyer should accept any other than an immune animal at any price."

Mr. Hunt has thus intentionally adopted a natural method of inoculation for practical purposes. An inspection of these farms in 1895 showed to me that the race-track and barn fields were not infected by ticks, while the creek-bottom pastures were. The practical value and wide application of this method in the South cannot be gainsaid. Mr. Hunt raises registered Jersey stock and markets them guaranteed against Southern fever. So long as possible customers retain ticks upon their farms, so long as is this his only method. It may well be followed by other cattlemen.

The only other means of producing disease in cattle and consequent immunity is that detailed by Dr. Smith:[1] "Another method of

[1] Texas Fever, p. 135.

inducing Texas fever is the injection of blood from cases of Texas fever. Such inoculations are apt to result in a mild attack if practised after the hot weather of midsummer." In a foot-note he states that "the Texas fever parasite was carried in the blood of a North Carolina animal three years after leaving the permanently infected territory." Since this was determined by inoculation and subsequent disease, it is evident that the blood could be drawn from healthy previously infected animals. Blood-inoculation experiments undertaken recently by the United States Department of Agriculture, in which the writer was detailed to take charge of the field portion, were more or less successful. In the event of complete success blood-inoculations can never become of wide use, for they are not so available or economic as other methods of attaining the same end. To illustrate: the only demand for it seems to be for inoculating breeding-cattle sent from the North. For best success a central vaccination-farm must be established, or cattle from infected localities taken to the farms where the operation is to be done. Then the blood is to be exchanged and the patient kept under observation for some weeks. All this would seem to require expert-work. The same result may be gained by procuring ticks from Southern infected farms, keeping them in a close-stoppered bottle until the young hatch from the eggs they lay, and then putting the seed-ticks upon the calves. The calves should be kept where the maturing ticks may not be able to infect others. It would be best to pick each as it matures. The adults would serve as material for new inoculations, providing the eggs hatch. About a month's time is required. The vaccination of older cattle is not yet warranted by results. If it is desirable to ship them into the infected territory, there would be no evil results provided they are so handled that they cannot become infected by ticks. The expense attendant upon handling cattle by these natural methods is inconsiderable as compared to that by artificial vaccination. The greatest recommendation of the latter is that, having an immune animal at hand, calves could be vaccinated at any time without risk to others. The risk by the natural method is inconsiderable and dependent alone upon the care of the operator.

Inoculation of Northern cattle is necessarily confined to a few high-priced breeding-animals. When Northern cattle are sent South without inoculation, kept upon uninfected farms, and their offspring intended for sale, the method detailed by Mr. Hunt must be followed. Blood-inoculations of young calves in the South can never become popular because of the certainty and cheapness of this method.

Isolation of cattle to prevent the spread of murrain has long been in vogue in the South by many of the more observant cattlemen. Having learned that the disease was carried from farm to farm, they simply stopped admitting strange cattle among their own or purchasing from places whence the cattle nearly always died. The shotgun policy of the West, by which cattlemen prevented the spread of Texas fever in earlier years, is not yet forgotten.

The pressing need of quarantine laws was learned by cattlemen early in the eighties, and through Congress carried into effect, being placed with the Bureau of Animal Industry. Though this law has been more or less imperfectly executed, from various causes, much good has been attained, especially in the handling of infected cattle consigned to the large markets for immediate slaughter. The result has been the almost complete prevention of loss among the susceptible cattle, which constitute the bulk of the trade.

There have annually been losses occurring in various States, even to this year, from violations of the law, cattle being shipped from infected localities through the quarantine, to be pastured or slaughtered at points where no quarantine station exists. Few if any prosecutions have been made in these cases under the federal law.

Early this year (1896) the State of Virginia enacted and passed a live-stock law which, in its coöperation with the federal law, has proved very effective. Where there existed during the previous years weekly violations of the federal quarantine laws and resulting losses from outbreaks, there has not been a single violation since. The few outbreaks that have occurred in that State have either been within the quarantine region or in isolated farms outside, where the ticks persisted from the year before and from which the disease was promptly eradicated through the action of State Veterinarian Dr. E. P. Niles. This example of the service of a good State law, efficient veterinarians, and the coöperation of stockowners illustrates what may be done throughout the extent of the line. Careful observation of the quarantine by railroad companies and stockmen has undoubtedly done more to keep disease from spreading than the inefficiently executed federal laws of previous years.

There is another direction in which greater effort may be made with good results. Cattle may now be taken from quarantine States, fed, watered, and rested *en route* as companies find convenient, and finally delivered to the consignee. Should the stops be where quarantine-pens are located all goes well; but these stations are comparatively few and far apart, and cattle are often unavoidably detained *en route*. To satisfy the requirements of the federal law, which requires unloading of cattle each twenty-eight hours on the road, the transportation companies are compelled to unload whenever they can. Outbreaks from these causes are, however, comparatively rare.

When the cattle are delivered, however, at the terminal station they become subject to local laws, and the federal government seems to lose all jurisdiction, and they are driven to pasture or to slaughter over highways, and often spread disease. To prevent these losses either the cattle should be excluded entirely from all places where suitable quarantine precautions could not be had, or sufficiently strict laws regulating the traffic should be made. In any event State authorities should be notified by the federal officers of the shipment of such cattle and their destination. When ticky cattle are sufficiently guarded at the terminal points as well as *en route* "Texas fever" will be unknown in the North.

Still another source of danger is from cattle driven on foot from one State or county to another across the quarantine line. Although this traffic is becoming less, it is still too frequent where public opinion is not educated in regard to the evil results to the cattle-trade of the uninfected regions, and also in States where the laws are inefficient. Strict maintenance of the quarantine along the three thousand miles of its extent can only be maintained by the coöperation of the people living along it, and this can only be gained by teaching them what the line is and what it is for. The highest duty of officers in such cases is as educators, not policemen. In view of the immense length of the line and the magnitude of the cattle-traffic, there is cause for wonderment that there are so few outbreaks from various causes.

Prevention of the tick-plague within the infected area has not been systematically undertaken in the past. It will more and more enter into the stockman's calculations until there is no longer need. The underlying principles of prophylaxy in the South consist in keeping cattle with ticks separate from others and their walks, in exterminating the ticks, and in immunizing calves as described. They are the same as for Northern localities, but must be thoroughly carried out. The tick is becoming a complete parasite in its habits, living as it does on cattle from the stage in which it emerges from the egg until it drops, completely mature, to lay other eggs, and, undergoing no or very little growth while off cattle, has become completely exposed to the attacks of man. There is no parasite which by intelligent effort can be so easily destroyed. Depending as it does on its immense fertility and the fortuitous return of the bovine host to the place where some other dropped its parent, every effort made by man to reduce the numbers of the adult parasites and the return of cattle to the infected places is met with the highest success in exterminating the pest. When once the cattleman understands that there can be no young ticks excepting as the parents are raised on his or someone's else cattle, and that cattle acquire them because they go where the old ticks have accidentally dropped, the question will be more than half met. To this must be added the fact that ticks do not lay eggs so long as they remain attached.

There are now many farms within the quarantine-area where there are no ticks and where the cattle are as susceptible to the disease as any in the North. To keep these free from disease it is only necessary to keep cattle with ticks entirely away from their pastures and walks, or to remove the ticks before adding them to the herd. Neither should they be sent to tick-infested farms. To keep disease from tick-infested farms, only immune cattle can be driven upon them. New purchases must be from other tick-infested farms or of young calves.

The method of preventing disease by inoculation has been given.

The radical treatment, the extermination of all ticks, is by far the best, most economical, and most effective method yet proposed. The amount of expenditure necessary to free any portion of the country would be more than met by the earnings to that community during the following year.

Until last year little had been attempted in this direction, excepting as farmers, disgusted at the appearance of the ticks, had continually picked them until all were gone. In 1892 Mr. H. J. Wing, dairyman of Georgia Experimental Station at Experiment, cleansed the station-farm by daily picking the ticks. Last year (1895) he supervised the cleansing of an adjoining farm, with the intention of preventing future outbreaks of the plague. Although eight head of cattle had died in 1894, susceptible cattle which were placed on the farm this year did not die, though exposed all summer to all pastures. On the station-farm he mingles cattle from all places, susceptible and unsusceptible, without loss, taking only the precaution of being sure that there are no ticks upon any new cattle when added to the herd. On adjoining farms there are annual losses.

During the last season dairy-herds near Manchester, Va., were kept cleaner of ticks than ever before by daily hand-picking alone, although they were driven upon the open lands where cattle of all kinds mingled. I believe that one of the tickiest farms there was entirely cleansed. The verification cannot be made until the coming season. Having left the work a little before the end of the tick-season leaves me in doubt as to the result.

For dairymen who may own as high as fifty or a hundred cattle the hand-picking method is all that is necessary, unless greasing be utilized to assist the method by keeping ticks off. For this purpose thorough rubbing in with a rag or brush of lard, fish-oil, cottonseed-oil, or West Virginia black oil as often as it is worn or washed off, is all that is necessary. Dr. M. Francis, of College Station, Texas, has been experimenting in dipping cattle in large vats for the removal of ticks from them. He finds the cottonseed-oil floating on water makes the best dip. This method has been extensively used in Queensland, Australia, where cattle are dipped before driving. The efficacy of the bland oils will be more in the preventing of ticks arriving on the cattle than in the destruction of those already on. In an experiment by the author, however, lard, when thoroughly rubbed upon young ticks, has not only stopped their growth, but caused them after some days to drop, without maturing.

The removal of ticks may be accomplished without either picking or greasing. This method has been described by Dr. Smith, and is but taking advantage of the life-history of the tick. If left alone, ticks mature and drop off. If no others are allowed to get upon them, they, of course, keep free. Dr. Smith recommended the placing of the tick-bearing cattle in an uninfected inclosure for fifteen days, then into a second for fifteen more. They should be examined closely, and, if free, put into the final pasturage. For the warm season the time is ample. A longer time may be necessary in colder weather, when the ticks do not mature so rapidly. On farms cultivated for varied crops this is the most satisfactory method, for there are always uninfected fields upon them. When every pasture-land is supposed to be infected, a portion may be abandoned or cultivated for a year, the cattle then cleaned of ticks, preferably in winter-time, and pastured upon them, leaving the remaining portion to become disinfected. It is possible that fields may become disinfected of seed-ticks in five months; but there is no positive evidence, so it is better to take a longer time. If hand-picking is resorted to in addition to turning on cultivated fields, the process is much shortened. If the fields are burned over about a month after the cattle are taken off, thus giving time for the young ticks to be hatched and to crawl upon the grasses, the cattle may be turned back upon them as soon as practicable. When this method is followed care must be taken to see that wet swails and resting-places of the cattle are also burned over. The burning will in any event diminish the number of ticks Barnyards and roadsides should be scraped or the latter burned over.

The ploughing of cultivated fields turns the ticks under and destroys them. The cattle can be pastured as soon thereafter as convenient or as soon as there is any fodder. In many counties of Virginia and North Carolina the practice of keeping cattle enclosed and changing them from field to field as the season affords food has entirely exterminated the ticks. Housing cattle nights and confining them during their noonday-rest in disinfected places will also keep off ticks.

Whatever the method adopted, thoroughness in its application until the task is accomplished is demanded. On most farms a combination of methods will be found most convenient. On few, excepting possibly the cattle-ranches, will the dipping-vats be found necessary. A little forethought and study in adapting the method to the surroundings will enable the cattle-owner to destroy the ticks with comparatively little expense. When it is remembered that ticks are scarce in spring and abundant later on, their destruction should be begun with the first seen and not given up until there are no more.

When the infected lands are completely cleared there can be no reason for quarantine. Though there may be infected lands which will produce the disease, a condition which has until now not been proved to exist, the disease cannot be carried to other cattle when the infected cattle are transported, except through inoculating agencies. The disease will then be confined to those localities which may be accurately mapped, and finally some means of extermination found. The facts now show that the greater part, if not all, of Southern lands are intermittently becoming infected and disinfected, and that infection follows ticky cattle.

Extermination of the pest is the only radical, rational method of treatment of the tick-plague. All other plans are but temporizing makeshifts, and in the end most expensive. Quarantine is at present but a necessary evil which will eventually become useless.

Vaccination, excepting of calves by natural methods for pasturing on infected lands, is comparatively useless, since the same results are permanently gained by destroying the ticks.

Future prophylactic work in the tick-plague lies in cleansing farms from ticks and contracting the line about the infected area until the country is cleansed.

